# Association between Microvolt T-Wave Alternans and Malignant Ventricular Arrhythmias in Chagas Disease

**DOI:** 10.5935/abc.20180056

**Published:** 2018-05

**Authors:** Bárbara Carolina Silva Almeida, André Assis Lopes do Carmo, Marco Paulo Tomaz Barbosa, José Luiz Padilha da Silva, Antonio Luiz Pinho Ribeiro

**Affiliations:** 1 Hospital das Clínicas e Faculdade de Medicina da Universidade Federal de Minas Gerais, Belo Horizonte, MG - Brasil; 2 Departamento de Estatística, Universidade Federal do Paraná, Curitiba, PR - Brasil

**Keywords:** Chagas Disease, Chagas Cardiomyopathy, Arrhythmias, Cardiac/complications, Defibrillators,Implantable, Death, Sudden, Cardiac

## Abstract

**Background:**

Sudden cardiac death is the most frequent death mechanism in Chagas disease, responsible for 55% to 65% of the deaths of patients with chronic Chagas cardiomyopathy (CCC). The most often involved electrophysiological mechanisms are ventricular tachycardia and ventricular fibrillation. The implantable cardioverter defibrillator (ICD) has a beneficial role in preventing sudden death due to malignant ventricular arrhythmias, and, thus the correct identification of patients at risk is required. The association of microvolt T-wave alternans (MTWA) with the appearance of ventricular arrhythmias has been assessed in different heart diseases. The role of MTWA is mostly unknown in patients with CCC.

**Objectives:**

To evaluate the association between MTWA and the occurrence of malignant ventricular arrhythmias in patients with CCC.

**Method:**

This is a case-control study including patients with CCC and ICD, with history of malignant ventricular arrhythmias (case group), and patients with CCC and no history of those arrhythmias (control group). The MTWA test results were classified as negative and non-negative (positive and indeterminate). The significance level adopted was a = 0.05.

**Results:**

We recruited 96 patients, 45 cases (46.8%) and 51 controls (53.1%). The MTWA test was non-negative in 36/45 cases (80%) and 15/51 controls (29.4%) [OR = 9.60 (95%CI: 3.41 - 27.93)]. After adjustment for known confounding factors in a logistic regression model, the non-negative result continued to be associated with malignant ventricular arrhythmias [OR = 5.17 (95%CI: 1.05 - 25.51)].

**Conclusion:**

Patients with CCC and history of malignant ventricular arrhythmias more often have a non-negative MTWA test as compared to patients with no history of arrhythmia.

## Introduction

Chagas disease remains a challenge of great importance in Brazil and Latin America, and is an emerging concern in North America and European countries.^1^ It is considered to be endemic in 21 countries, infects 6 to 7 million people worldwide,^2^ accounting for the death of around 12,000 patients per year.^3^

Chronic Chagas cardiomyopathy (CCC) is the most important presentation of Chagas disease, because of its high frequency, severity and great impact on morbidity and mortality. Chronic Chagas cardiomyopathy has a wide range of manifestations, such as heart failure, conduction blocks, thromboembolic events and sudden death.^4^^,^^5^ Sudden death is the most common mechanism of death of those patients, occurs in the presence or absence of advanced heart disease, and can be the first manifestation of the disease. The electrophysiological mechanisms most frequently involved are the ventricular arrhythmias: sustained ventricular tachycardia and ventricular fibrillation.^4^^,^^6^

Implantable cardioverter-defibrillator (ICD) has a great impact on the prevention of sudden death due to malignant ventricular arrhythmias.^7^^,^^8^ The use of the ICD in secondary prevention is well accepted in CCC, despite the lack of large studies, based on the results obtained from other populations.^7^^,^^9^ However, its use in primary prevention is still controversial because of the high cost, intrinsic risks in implantation, and adverse effects.^10^^,^^11^ Therefore, identifying patients with CCC at risk for sudden death due to malignant ventricular arrhythmias is necessary.

The microvolt T-wave alternans (MTWA) test is a non-invasive test associated with the appearance of ventricular tachyarrhythmias assessed in different clinical conditions with a high negative predictive value to identify patients at risk.^12^^-^^17^ That test recognizes fluctuations of the T-wave morphology and amplitude beat to beat, measured in microvolts. Those fluctuations reflect space-temporal heterogeneity of ventricular repolarization, which is considered a predisposing condition to the beginning and perpetuation of ventricular arrhythmias.

It is worth noting the association of MTWA with malignant arrhythmias in several clinical conditions, but few studies have included patients with CCC. This study was aimed at assessing the possible association between MTWA and malignant ventricular arrhythmias in Chagas disease.

## Method

### Study

This is an observational, case-control study, approved by the Ethics Committee in Research of the Federal University of Minas Gerais (COEP 7918/12). The patients were recruited between 2011 and 2014.

### Patients

The sample consists of patients diagnosed with CCC being followed up at the Hospital das Clínicas of the Federal University of Minas Gerais (HC-UFMG). The individuals agreed to participate and provided written informed consent. Patients should be older than 18 years, have a positive serology for Chagas disease and meet all the diagnostic criteria for CCC, which include asymptomatic structural heart disease with typical electrocardiographic changes,^18^ or heart failure with preserved or reduced left ventricular ejection fraction (LVEF), with current or previous symptoms.

The case group consisted of patients with CCC and history of malignant ventricular arrhythmia, with indication for ICD implantation for secondary prophylaxis and authorization issued by the High-Complexity Commission of the Brazilian Unified Health System (SUS), according to the ordinance # 152, of March 8, 2007,^19^ updated by the ordinance # 1, of January 2, 2014.^20^ Patients with CCC and no previous history of malignant ventricular arrhythmia comprised the control group. 

According to the ordinance # 152, of March 8, 2007, the major indications for ICD implantation in Brazil are as follows:^19^

- Individuals resuscitated from documented cardiac arrest due to tachycardia or ventricular fibrillation of non-reversible cause, with LVEF ≤ 35% or structural heart disease;
- Spontaneous, sustained ventricular tachycardia, of non-reversible cause, with LVEF ≤ 35%;
- On the electrophysiological study, syncope of undetermined etiology with induction of hemodynamically unstable sustained ventricular tachycardia, or clinically relevant ventricular fibrillation with LVEF ≤ 35% or structural heart disease.

Individuals with the following characteristics were excluded from the study: difficulty to walk on the treadmill; NYHA functional class IV heart failure; atrial fibrillation or flutter; pacemaker dependency. In addition, individuals with absolute contraindications to undergo exercise test, such as cardiac arrhythmias leading to hemodynamic instability, decompensated heart failure and acute non-cardiac conditions that could be aggravated by physical exercise, were excluded from the study.^21^

### Microvolt T-wave alternans test

The individuals included in this study underwent a medical interview with a standard questionnaire, physical examination and transthoracic echocardiography. Left ventricular ejection fraction was calculated by use of the Simpson's method. Later, the patients underwent the MTWA test, at the ergometry sector of the Hospital das Clínicas of the UFMG.

For performing the MTWA test, the following items were used: Micro-V Alternans Sensors^TM^ of Cambridge Heart high-resolution electrodes, which minimize noise and artifacts; the Cambridge Heart - HearTwave software for analysis and report; and a treadmill. Chronically used medications were maintained.

The MTWA test consists in proper preparation with skin cleansing and removal of the superficial layer of dead cells by use of abrasion, placement of electrodes in the 12 standard electrocardiographic leads and in the 3 orthogonal leads (X, Y and Z).

Data from the electrocardiographic tracing were collected at rest, during exertion on the treadmill, and during the recovery phase. During exertion, the patient should reach a heart rate between 100 and 110 beats per minute (bpm) and sustain it for 2 minutes and 30 seconds. Then, heart rate between 110 and 120 bpm should be reached and sustained for 1 minute and 30 seconds. For the test to be considered valid, target heart rate should be maintained for at least 60% of the determined time period.

The software provides an analysis with measurement of MTWA, characterizing the test as positive, negative or indeterminate. The positive test consists in T-wave alternans with amplitude ≥ 1.9 µV sustained for at least 1 minute, with an initial heart rate < 110 bpm or at rest, in an orthogonal lead or two adjacent precordial leads. The negative test does not detect any significant T-wave alternans for 1 minute with a heart rate ≥ 105 bpm, if there is no impairment to the tracing due to noise or more than 10% of ectopic beats.^22^^,^^23^ The tests that do not meet any of those criteria are considered indeterminate. The indeterminate tests attributed to noise were repeated. Then the tests were grouped as negative or non-negative (positive and indeterminate), based on studies about the impact of the indeterminate test on the outcome of ventricular arrhythmias. An indeterminate test due to patient's factors, such as impossibility to keep heart rate between 105 and 110 bpm, frequent extrasystoles and MTWA not sustained for 1 minute, is associated with the occurrence of ventricular arrhythmias similarly to the way the positive test is.^24^

### Sample calculation

The sample was calculated with the Power and Sample Size Calculations software.^25^ Considering that Barbosa et al.^26^ have found non-negative results in 81.8% of the Chagas disease patients wearing an ICD, estimating that those without malignant ventricular arrhythmia would have 30% less non-negative MWTA tests (57%), for a power of 80% and alpha error of 5%, we found 50 patients in each group.

### Statistical analysis

Initially, the case and control groups were compared regarding their clinical characteristics by use of Fisher exact test. The variables tested were sex, age (older or younger than 60 years), reduced or preserved LVEF, and beta-blocker use. There was a significant disparity between the groups, and to assess the association between MTWA and the occurrence of malignant ventricular arrhythmias, multiple logistic regression models were adjusted, including the potential confounding covariables. The covariables age and LVEF were entered into the model continuously. The model calibration was assessed by use of Hosmer-Lemeshow test. The results were expressed as odds ratio (OR) with its respective confidence interval. The significance level adopted was α = 0.05. All analyses were performed with the R statistical software, 3.3.2 version.^27^

## Result

This study recruited 96 patients with CCC as follows: 45 patients (46.8%) with an ICD, constituting the case group; and 51 (53.1%) without an ICD and no known history of ventricular arrhythmia, constituting the control group. Table 1 describes the sample. Of the total sample, 48 patients (50%) were of the male sex, 42.2% of the case group and 53.1% of the control group, p = 0.220. Of the patients with an ICD, 57.8% were older than 60 years, while of those with no ICD, only 1.96% were older than 60 years, p < 0.001. Of the total sample, 37 patients had reduced LVEF (38.5%), 31 patients (68.9%) in the case group, and 6 patients (19.6%) in the control group, p < 0.001. In addition, the distribution of beta-blocker users was as follows: 37 patients in the case group (82.2%), and 10 patients in the control group (19.6%), p < 0.001.

**Table 1:** Characteristics of the sample.

	All (96)	Case group (45)	Control group (51)	p
Number of patients	96	45	51	-
Male sex *	48	19	29	0.220
Mean age (years)	55	62	49	-
Age > 60 years *	27	26	1	< 0.001
Mean ejection fraction (%)	48,8	39	58	-
Reduced ejection fraction (< 45%) *	37	31	6	< 0.001
Beta-blocker use *	47	37	10	< 0.001

* Number of patients

The MTWA test had a non-negative result in 51 patients (53.1%) as follows: 36/45 patients (80%) in the case group and 15/51 patients (29.4%) in the control group, OR = 9.60 (95%CI: 3.41 - 27.93). Because of the difference in characteristics between the groups, a logistic regression model was created to correct the disparities between them, including age, sex, LVEF and beta-blocker use. Table 2 shows the results of data analysis.

**Table 2:** Factors related to the presence of ventricular arrhythmias in the multivariate logistic regression model.

	p	OR	95%CI LL	95%CI UL
MWTA	0.044	5.17	1.05	25.51
Beta-blocker	0.139	3.73	0.65	21.40
Sex	0.118	0.27	0.05	1.39
LVEF	0.011	0.91	0.85	0.98
Age	0.005	1.13	1.04	1.22

LL: lower limit; UL: upper limit; MWTA: microvolt T-wave alternans; LVEF: left ventricular ejection fraction.

The model showed that the difference is statistically significant between the case and control groups regarding the result of the MTWA test [OR = 5.17 (95%CI: 1.05 - 25.51)]. The Hosmer-Lemeshow test showed good calibration of the model (p = 0.872).

## Discussion

In this case-control study with adjustments for other significant variables, we observed the association between the non-negative result of the MTWA test and the occurrence of ventricular tachyarrhythmias in patients with CCC, with OR = 5.17 (95%CI: 1.05 - 25.51), suggesting that MTWA may play a role in the assessment of the risk for sudden death of patients with Chagas heart disease.

The occurrence of ventricular tachyarrhythmias seems more common in Chagas disease than in heart diseases of other etiologies.^4^ However, there is neither a method nor a score to properly identify patients at risk for sudden death due to those arrhythmias.

The MTWA test has been widely studied in heart diseases of several etiologies, and countless studies have evidenced the association between the non-negative result of the test and the occurrence of malignant ventricular arrhythmias.^13^^-^^17^ The present study corroborates previous studies from our search group that have suggested a role for MTWA in the stratification of risk for sudden death in CCC.

Initially, Ribeiro et al. have observed that the T-wave amplitude variability measured in 11-minute high-resolution ECG tracings - a phenomenon analogous to MTWA - related to higher risk of death in patients with CCC after following 113 patients up at an outpatient clinic for 106 months [HR = 5.76 (95%CI: 1.31-25.23)].^28^ In a subsequent study, Raadschilders et al. have demonstrated a higher occurrence of non-negative MTWA test among patients with CCC as compared to individuals with Chagas disease but no heart impairment and patients with negative serology for Chagas disease.^29^ Barbosa et al., performing the test in patients with indication for ICD implantation and diagnosed with Chagas heart disease and heart diseases of other etiologies, have assessed the association between MTWA and the occurrence of the outcomes 'proper therapy' and 'death'. Those authors have concluded that there is a relationship between a non-negative (positive and indeterminate) MTWA test and higher occurrence of proper therapy during the follow up of patients with Chagas disease, which was not observed among patients with heart disease of other etiologies. For patients with CCC, the test had sensitivity and negative predictive value of 100%.^26^

The higher occurrence of an altered MTWA test in CCC can be explained by the inflammatory and fibrosing nature of the disease. Chagas heart disease is a chronic myocarditis, with damage to the tissue of the cardiac chambers and conduction system.^30^ The destruction of cardiomyocytes and the resulting fibrosis cause architectural myocardial disarray, which can result in intercellular decoupling. This decoupling could cause a variability in cardiomyocyte membrane repolarization due to the difference in duration of their action potentials. Therefore, myocardial zones refractory to depolarization appear, tending to divide the depolarization current, the mechanism by which the variability would be linked to arrhythmogenesis, favoring conduction blocks and reentry induction.^31^

The spatial heterogeneity of ventricular repolarization is considered a predisposing condition to initiate and perpetuate ventricular arrhythmias. That heterogeneity can be measured by use of the MTWA test, which would justify finding more changes in the MTWA test of patients with CCC and previous history of malignant arrhythmias.

The MTWA test has difficulties related to the high cost of high-resolution electrodes and its own performance. Many individuals submitted to the test cannot reach and sustain the heart rate required or cannot undergo the exertion phase on the treadmill. The amount of indeterminate results due to noise or early interruption because of the patient's conditions are also a limiting factor. In addition, the result is classified qualitatively, which can be considered another limitation.

This study has limitations related partially to its observational, case-control design. The number of patients found for the case group was 45, not the 50 predicted in the sample calculation. The case group, defined by a previous history of malignant arrhythmias and indication for ICD, had a greater number of patients with reduced LVEF, of beta-blocker users and of patients with more advanced age. This is justified by the inclusion criterion in the group, because the patients with reduced LVEF would be more predisposed to develop ventricular arrhythmias. In addition, according to the 2007 ordinance,^19^ patients with LVEF < 35% have an indication for priority to undergo ICD implantation. A logistic regression model was created to correct the disparity between the groups, maintaining the association between non-negative test and the occurrence of arrhythmias. The model may, however, not have corrected all differences between patients. Nevertheless, the large proportional difference of non-negativity between the case and control groups, corroborated by the magnitude of the association obtained on logistic regression, suggest that the phenomenon observed is real and significant.

## Conclusion

This study assessed the presence of MTWA in patients with CCC and previous history of malignant ventricular arrhythmias and in patients with no previous history of those arrhythmias. The association between non-negativity of the MTWA test and the occurrence of malignant ventricular arrhythmias in CCC was evidenced. Further assessment in a prospective study is required to establish the causality and clinical application of the test in those patients.

## References

[1] Schmunis GA, Yadon ZE (2010). Chagas disease: A Latin American health problem becoming a world
health problem. Acta Trop.

[2] World Health Organization Chagas disease (American trypanosomiasis): Fact sheet.

[3] World Health Organization Neglected, Tropical and Vector Borne Diseases: Chagas Disease.

[4] Biolo A, Ribeiro AL, Clausell N (2010). Chagas cardiomyopathy-where do we stand after a hundred
years. Prog Cardiovasc Dis.

[5] Ribeiro AL, Nunes MP, Teixeira MM, Rocha MO (2012). Diagnosis and management of Chagas disease and
cardiomyopathy. Nat Rev Cardiol.

[6] Bestetti RB, Cardinalli-Neto A (2008). Sudden cardiac death in Chagas' heart disease in the contemporary
era. Int J Cardiol.

[7] Connolly SJ, Hallstrom RC, Cappato R, Schron EB, Kuck KH, Zipes DP (2000). Meta-analysis of the implantable cardioverter defibrillator
secondary prevention trials. AVID, CASH and CIDS studies. Antiarrhythmics vs
Implantable Defibrillator study. Cardiac Arrest Study Hamburg . Canadian
Implantable Defibrillator Study. Eur Heart J.

[8] Moss AJ, Zareba W, Hall WJ, Klein H, Wilber DJ, Cannom DS, Multicenter Automatic Defibrillator Implantation Trial II
Investigators (2002). Prophylactic implantation of a defibrillator in patients with
myocardial infarction and reduced ejection fraction. N Engl J Med.

[9] Ezekowitz JA, Armstrong PW, McAlister FA (2003). Implantable cardioverter defibrillators in primary and secondary
prevention: a systematic review of randomized, controlled
trials. Ann Intern Med.

[10] Rosenqvist M, Beyer T, Block M, den Dulk K, Minten J, Lindemans F (1998). Adverse events with transvenous implantable
cardioverter-defibrillators: a prospective multicenter study. Circulation.

[11] Poole JE, Johnson GW, Hellkamp AS, Anderson J, Callans DJ, Raitt MH (2008). Prognostic importance of defibrillator shocks in patients with
heart failure. N Engl J Med.

[12] Rosenbaum DS, Jackson LE, Smith JM, Garan H, Ruskin JN, Cohen RJ (1994). Electrical alternans and vulnerability to ventricular
arrhythmias. N Engl J Med.

[13] De Ferrari GM, Sanzo A (2009). T-wave alternans in risk stratification of patients with
nonischemic dilated cardiomyopathy Can it help to better select candidates
for ICD implantation?. Heart Rhythm.

[14] Salerno-Uriarte JA, De Ferrari GM, Klersy C, Pedretti RF, Tritto M, Sallusti L, ALPHA Study Group Investigators (2007). Prognostic value of T-wave alternans in patients with heart
failure due to nonischemic cardiomyopathy: results of the ALPHA
study. J Am Coll Cardiol.

[15] Chow T, Kereiakes DJ, Bartone C, Booth T, Schloss EJ, Waller T (2007). Microvolt T-wave alternans identifies patients with ischemic
cardiomyopathy who benefit from implantable cardioverter-defibrillator
therapy. J Am Coll Cardiol.

[16] Tanno K, Ryu S, Watanabe N, Minoura Y, Kawamura M, Asano T (2004). Microvolt T-wave alternans as a predictor of ventricular
tachyarrhythmias a prospective study using atrial pacing. Circulation.

[17] Gehi AK, Stein RH, Metz LD, Gomes JA (2005). Microvolt T-wave alternans for the risk stratification of
ventricular tachyarrhythmic events: a meta-analysis. J Am Coll Cardiol.

[18] Andrade JP, Marin-Neto JA, Paola AA, Vilas-Boas F, Oliveira GM, Bacal F, Sociedade Brasileira de Cardiologia (2011). [I Latin American guidelines for the diagnosis and
treatment of Chagas cardiomyopathy]. Arq Bras Cardiol.

[19] Brasil, Ministério da Saúde, Secretaria de Atenção à Saúde Portaria nº. 152, de 8 de março de 2007. Define
através da Política Nacional de Atenção
Cardiovascular de Alta Complexidade, o Implante de Marcapassos de Alto
Custo. Diário Oficial da União. Poder Executivo. Brasilia
(DF); 09 de março de 2007.

[20] Brasil, Ministério da Saúde, Secretaria de Atenção à Saúde Portaria nº. 1, de 2 de Janeiro de 2014. Atualiza o protocolo do
uso do cardioversor desfibrilador implantável a ser adotado nos
estabelecimentos de saúde credenciados no Sus. Diário Oficial
da União.Poder Executivo. Brasilia(DF); 03 de janeiro de 2014.
Seção 1,p.29-32.

[21] Fletcher GF, Ades PA, Kligfield P, Arena R, Balady GJ, Bittner VA, American Heart Association Exercise, Cardiac Rehabilitation, and
Prevention Committee of the Council on Clinical Cardiology, Council on
Nutrition, Physical Activity and Metabolism, Council on Cardiovascular
and Stroke Nursing, and Council on Epidemiology and Prevention (2013). Exercise standards for testing and training: a scientific
statement from the American Heart Association. Circulation.

[22] Merchant FM, Salerno-Uriarte JA, Caravati F, Falcone S, Molon G, Marangoni D (2015). Prospective use of microvolt T-wave alternans testing to guide
primary prevention ICD therapy. Circ J.

[23] Kraaier K, Olimulder MA, van Dessel PF, Wilde AA, Scholten MF (2014). Prognostic value of microvolt T-wave alternans in a real-world
ICD population. Twente ICD Cohort Studie (TICS). Neth Heart J.

[24] Kaufman ES, Bloomfield DM, Steinman RC, Namerow PB, Costantini O, Cohen RJ (2006). "Indeterminate" microvolt T-wave alternans tests predict high
risk of death or sustained ventricular arrhythmias in patients with left
ventricular dysfunction. J Am Coll Cardiol.

[25] Chow S, Shao J, Wang H (2008). Sample Size Calculations in Clinical Research.

[26] Barbosa MP, da Costa Rocha MO, Neto ES, Brandão FV, Lombardi F, Ribeiro AL (2016). Usefulness of microvolt T-wave alternans for predicting outcome
in patients with Chagas disease with implantable cardioverter
defibrillators. Int J Cardiol.

[27] R Core Team (2016). A language and environment for statistical computing.

[28] Ribeiro AL, Rocha MO, Terranova P, Cesarano M, Nunes MD, Lombardi F (2011). T-wave amplitude variability and the risk of death in Chagas
disease. J Cardiovasc Electrophysiol.

[29] Raadschilders L, Barbosa MP, Carmo AA, Nouwen JL, Rocha MO, Ribeiro AL (2015). Microvolt T-wave alternans in Chagas disease. Int J Cardiol.

[30] Nunes MC, Dones W, Morillo CA, Encina JJ, Ribeiro AL, Council on Chagas Disease of the Interamerican Society of
Cardiology (2013). Chagas Disease: an overview of clinical and epidemiological
aspects. J Am Coll Cardiol.

[31] Wilson LD, Rosenbaum DS (2007). Mechanisms of arrythmogenic cardiac alternans. Europace.

